# H3G34-Mutant Gliomas—A Review of Molecular Pathogenesis and Therapeutic Options

**DOI:** 10.3390/biomedicines11072002

**Published:** 2023-07-15

**Authors:** Anthony V. Nguyen, Jose M. Soto, Sarah-Marie Gonzalez, Jennifer Murillo, Eric R. Trumble, Frank Y. Shan, Jason H. Huang

**Affiliations:** 1 Department of Neurosurgery, Baylor Scott and White Medical Center, Temple, TX 76508, USA; anthony.nguyen2@bswhealth.org (A.V.N.); jose.soto@bswhealth.org (J.M.S.); sarah.gonzalez1@bswhealth.org (S.-M.G.); jennifer.murillo@bswhealth.org (J.M.); eric.trumble@bswhealth.org (E.R.T.); yuan.shan@bswhealth.org (F.Y.S.); 2 Department of Neurology, Baylor Scott and White Medical Center, Temple, TX 76508, USA; 3 Department of Pathology, Baylor Scott and White Medical Center, Temple, TX 76508, USA; 4 Department of Surgery, Texas A&M University College of Medicine, Temple, TX 76508, USA

**Keywords:** neuro-oncology, neurosurgical oncology, neurosurgery, glioma, diffuse hemispheric glioma, H3G34, epigenetics, molecular biology

## Abstract

The 2021 World Health Organization Classification of Tumors of the Central Nervous System reflected advances in understanding of the roles of oncohistones in gliomagenesis with the introduction of the H3.3-G34R/V mutant glioma to the already recognized H3-K27M altered glioma, which represent the diagnoses of pediatric-type diffuse hemispheric glioma and diffuse midline glioma, respectively. Despite advances in research regarding these disease entities, the prognosis remains poor. While many studies and clinical trials focus on H3-K27M-altered-glioma patients, those with H3.3-G34R/V mutant gliomas represent a particularly understudied population. Thus, we sought to review the current knowledge regarding the molecular mechanisms underpinning the gliomagenesis of H3.3-G34R/V mutant gliomas and the diagnosis, treatment, long-term outcomes, and possible future therapeutics.

## 1. Introduction

The estimated incidence of malignant brain and central nervous system (CNS) tumors in the United States is 7.08 cases per 100,000 people per year, and malignant CNS tumors are the leading cause of cancer-related deaths in patients aged 0–14 years [1]. Gliomas are estimated to compromise 80% of malignant CNS tumors [1]. In 2021, The World Health Organization (WHO) Classification of Tumors of the CNS was updated due to recent advances in genetic sequencing and the identification of molecularly distinct entities; some tumors that would have previously been diagnosed as glioblastoma multiforme (GBM) were re-classified. A diagnosis of “diffuse hemispheric glioma, H3G34-mutant” (DHG) was delineated in the pediatric-type diffuse high-grade glioma category. These patients have a median overall survival (OS) of 18–22 months and median age at diagnosis of 15–16 years, which is different from the general GBM population [2,3]. DHG is frequently diagnosed in adult patients as well [4,5].

The histone variant H3.3 glycine 34 to arginine/valine (H3.3-G34R/V) mutation was first recognized in 2012 in what were considered pediatric GBM cases [6,7,8,9]. This diagnostic entity is thus younger than the Stupp protocol of concurrent chemoradiation followed by adjuvant temozolomide monotherapy [10] that has long been the standard of care for adult GBM cases, and advances in the understanding of H3G34-mutant gliomas are still being made. In the years following the recognition of histone mutations in pediatric-type high-grade gliomas (HGG), it was realized that this would become a distinct diagnostic entity due to differences in epigenetics, transcriptomes, proteomes, associated characteristics such as an older age at the onset of DHG, anatomic distribution, and histopathological and radiological features [11,12,13,14]. Prior to the identification of H3G34-mutant DHG as a distinct clinical entity, patients with H3G34-mutant gliomas were often diagnosed with a primitive neuroectodermal tumor (PNET) or GBM [3,15]. This resulted in the heterogenous treatment of patients with what is now recognized as one tumor type, including craniospinal radiation and differing chemotherapy regimens [15]. Furthermore, studies have demonstrated that when adult GBM therapeutic interventions are applied in the pediatric population, there is little-to-no improvement in survival, indicating that this disease entity needs its own avenues of research [16]. We thus aimed to summarize the relevant biochemical pathways, pathogenesis, and therapeutic options of H3G34-mutant gliomas.

## 2. Methods

The authors performed a literature search of the PubMed and OVID databases on 6 April 2023, utilizing the search terms “H3 G34*” OR “H3G34*” OR “H3-G34*” OR “H3F3A G34*” OR “H3F3AG34*” OR “H3F3A-G34*” OR “H3.3 G34*” OR “H3.3G34*” OR “H3.3-G34*”. All articles published in English focusing on glioma were reviewed in order to compile and synthesize information regarding the underlying genetic alterations, relevant molecular pathways, pathogenesis, clinical manifestations, and treatment of H3G34-mutant gliomas. We excluded articles that primarily investigated a giant cell tumor of the bone, which is also known to be associated with histone H3 mutations [17]. Studies that investigated the molecular genetics and epigenetics relevant to H3-G34 mutant gliomas were included, even if the primary focus was on other malignancies. Reviews and summaries of primary molecular studies that did not conduct any novel experiments were reviewed but were not included in the synthesis of relevant molecular pathways, clinical features, and possible therapeutics of/for H3.3-G34 mutant gliomas. Additionally excluded were articles that grouped H3.3-G34 mutant gliomas with other high-grade gliomas in descriptive studies without reporting results on this subgroup separately. Articles that focused on radiologic features or patient treatments and outcomes were used as a source for a secondary literature search. If they conducted a systematic review, the articles identified and included in the review were included as articles identified in a secondary literature review. We also herein include hypotheses regarding the tumorigenesis of H3.3-G34R/V by analyzing the implicated pathways involved.

## 3. Literature Review

**Figure 1:**
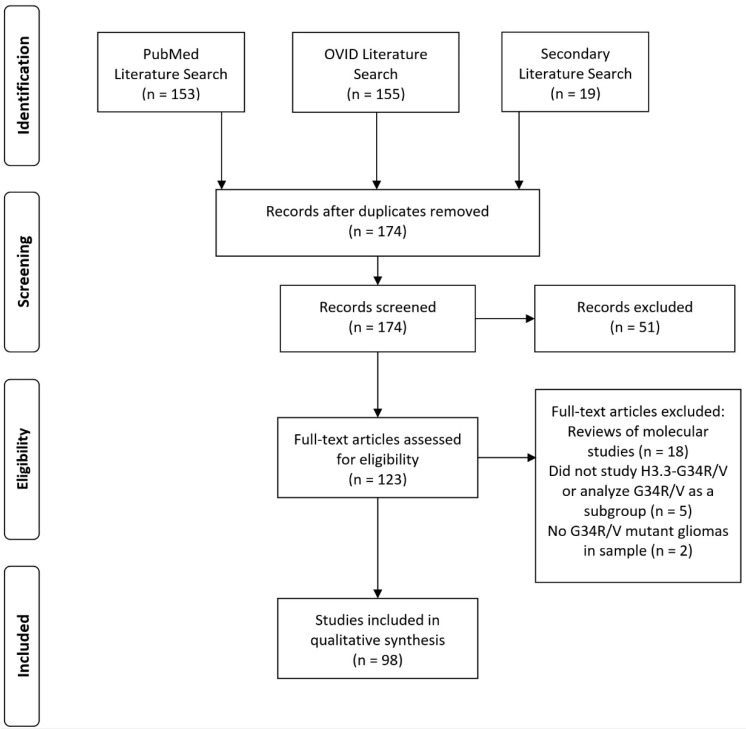
Flow diagram demonstrating the number of studies that were identified in the literature search, screened, excluded, and included in the final analysis.

**Figure 2:**
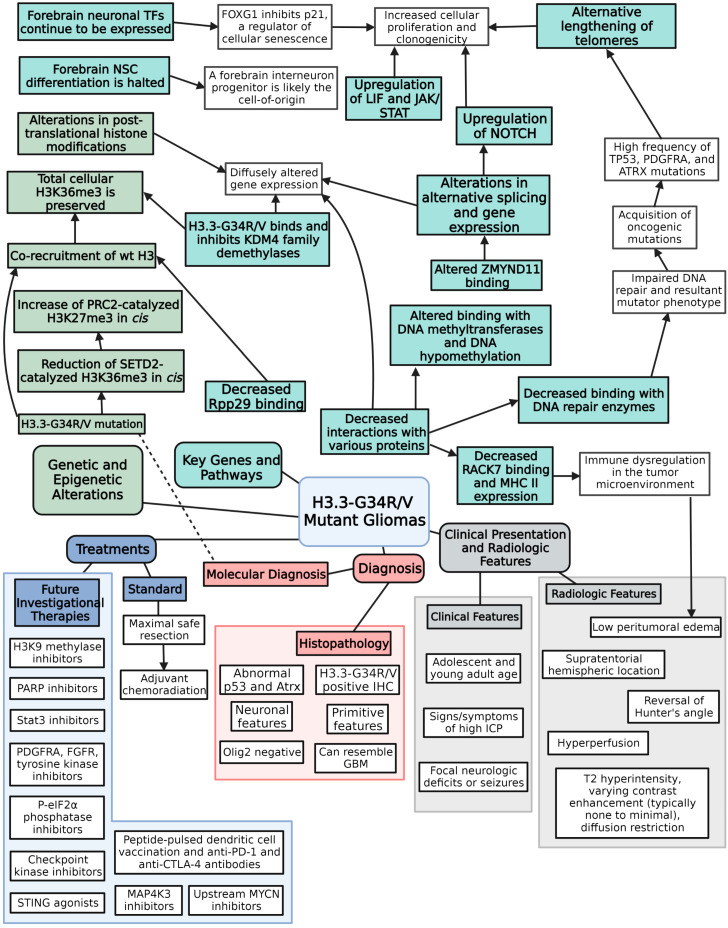
Schematic depicting main key epigenetic alterations, genes and molecular pathways, clinical presentation and radiologic features, diagnostic criteria, and current and experimental/proposed therapies related to H3.3-G3R/V mutant gliomas.

After the consideration of inclusion and exclusion criteria, 98 studies were included in the synthesized review. The flowsheet of study inclusion and exclusion can be found in Figure 1. These studies are intermixed with other articles that provide supplementary information. The main key epigenetic alterations, genes and molecular pathways, clinical presentation and radiologic features, diagnostic criteria, and current and experimental/proposed therapies are summarized in Figure 2.

### 3.1. Brief Overview of Epigenetics

Deoxyribonucleic acid (DNA) is considered to be the universal genetic code that carries the information needed to create living organisms. The central dogma of biology explains how DNA becomes a living organism, and that is that DNA is transcribed into ribonucleic acid (RNA), which is then translated into proteins. Proteins are highly variable and versatile and can serve numerous functions including structural support, molecular transport, and the facilitation of biochemical reactions in order to convert molecules to other molecules necessary for supporting cell life [18].

The amount of information that must be encoded to create a human is considerable. In order to package this information into the nucleus of a cell, DNA is organized into a nucleosome, a tightly wound structure of DNA and histones, proteins that organize DNA and facilitate its accessibility [19]. The phosphodiesterase backbone of deoxyribonucleic acid (DNA) is negatively charged due to the abundance of oxygen atoms. Histones typically have alkaline, positively charged amino acid side chains that are capable of interacting with the negatively charged DNA. A nucleosome consists of 146 base pairs of DNA wound around a histone octamer of two copies of H2A, H2B, H3, and H4 [19]. Histone H3 has several different variants, some of which are expressed during particular cases of the cell cycle, are only expressed in certain regions of chromosomes, or are only expressed in certain tissues. However, H3.3 is a variant that is constitutively expressed and diffusely present [20]. H3.3 production is encoded for by the genes H3F3A and H3F3B, and the variant differs from other H3 variants by several amino acids that regulate chaperone protein affinity and chromatin expression [20].

Histones are heavily modified in order to regulate DNA expression, and the best understood modifications are acetylation, methylation, phosphorylation, and ubiquitination/ubiquitylation [21]. Acetylation adds a hydrophobic carbon chain, and phosphorylation adds a negatively charged molecule to the side chain; both typically serve to decrease the electrostatic attraction between histones and DNA, making DNA more accessible to other proteins such as DNA transcriptase. Methyl groups are small while ubiquitin is a peptide chain of 76 amino acids, much larger than even phosphate groups. Methylation and ubiquitination can be performed in various degrees (mono- or poly-) and have varying effects on DNA expression [21,22]. These modifications are referred to as epigenetics as they affect DNA regulation and result in phenotypic variations without changing the sequence of DNA. Epigenetic alterations are known to be involved in oncogenesis and have therapeutic implications as well [23,24].

### 3.2. Histone H3.3-G34R/V Mutations

A mutation of histone variant H3.3 where the 27th residue, a lysine side chain, was replaced by methionine was discovered to be the driving mutation for diffuse midline glioma (DMG) [6], a distinct clinical identity once thought to represent a phenotype of pediatric GBM. A different mutation of the H3F3A gene in which glycine 34 was replaced by arginine or valine (H3F3A-G34R/V) was found to be the key mutation in patients with DHG, also referred to as non-brainstem pediatric GBM in the literature [7,17]. Substitutions of G34 with tryptophan or leucine, on the other hand, are associated with giant cell tumors of the bone. This review herein focuses on H3F3A-G34R/V and DHG. 

**Figure 3:**
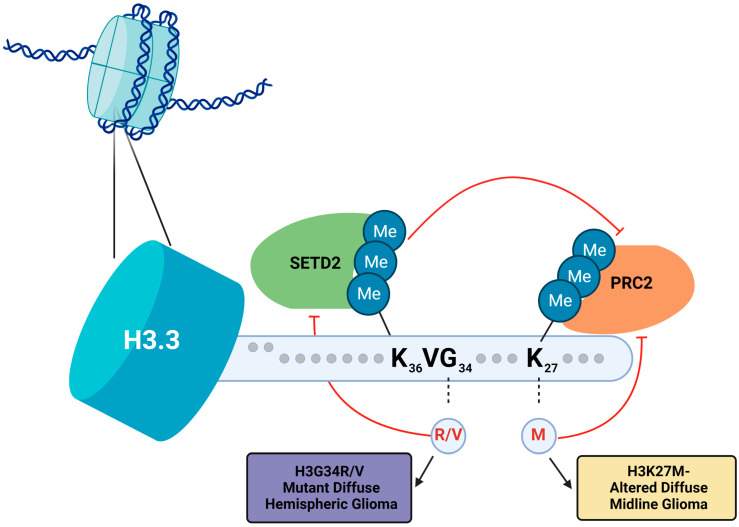
Diagram of the side chains of the H3.3 histone variant N-terminus following initiator methionine cleavage. Depicted are the H3K27M mutation, its inhibition of PRC2 (and thus inhibition of trimethylation of H3K27), as well as the H3G34 mutations, including the effects of G34R/V on inhibition of SETD2 and catalyzation of trimethylation of H3K36.

H3G34 mutations that result in gliomas exclusively affect histone variant H3.3 on the H3F3A gene and more frequently involve arginine than valine substitutions [2]. The tumorigenic mutation is additionally mutually exclusive with the pathogenic H3K27M oncohistone mutation [2]. While H3G34 is not a target for post-translational modifications (PTM) of histones in the regulation of the epigenome (i.e., H3G34 is not methylated) [20], it exists in close proximity to K36, and trimethylation of H3K36 (H3K36me3) is associated with actively transcribed euchromatin [20]. However, the effects of H3K36me3 may be context-dependent and are numerous, including the regulation of gene expression, transcription elongation, alternative splicing, and DNA repair [25]. Figure 3 summarizes the N-terminus sequences of H3.3, the oncogenic histone mutations, and its immediate effects on the post-translational modification of nearby side chains.

### 3.3. Histone H3.3-G34R/V Mutations Affect Post-Translational Modification of H3K36 and H3K27

Studies have demonstrated that the replacement of the small glycine side chain at residue 34 of H3.3 with larger side chains results in decreased SETD2 activity [26,27]. SETD2 catalyzes the methylation of H3K36 along with NSD1, NSD2, and ASH1L. However, of these enzymes, SETD2 is the only one that can fully modify H3K36 to a trimethylated state [26], and H3K36me3 is associated with euchromatin as mentioned above. H3F3A-G34R/V mutations are thought to affect SETD2 binding for the enzymatic conversion of H3K36 to a trimethylated state as the 33rd and 34th residues (which are both glycine in the wild-type H3.3 variant) lying in a constrained substrate channel of SETD2. The replacement of glycine with a bulkier side chain results in steric clashing with F1668 and Y1671 of SETD2 and a lower enzyme affinity [27,28,29]. This results in decreased H3K36me3 in *cis* (on the same histone harboring the mutation), but overall cellular H3K36me3 levels are preserved [9,26,27,28,30,31,32,33,34]. As the SETD2-mediated effect only occurs in *cis*, this does not explain the dominant effect of the H3.3-G34R/V mutant. Additionally, Fontebasso et al. demonstrated that HGGs with SETD2 mutations exhibited different DNA methylation signatures than that of H.3-G34R/V mutant gliomas [35], and Yadav et al. demonstrated that the deletion of the yeast homolog of SETD2 did not result in similar gene expression when compared to H3.3-G34R/V mutations [36].

One possible explanation for the preservation of total H3K36me3 levels even in the presence of decreased H3K36me3 in *cis* is that the recruitment of H3.3-wild-type (wt) to chromatin could be upregulated in G34R/V mutants [30]. Data suggest that this could be mediated by Rpp29, an RNase P subunit, which has a decreased binding affinity to H3.3-G34R [37]. Rpp29-ko resulted in increased levels of H3K36me3. Lim et al. did demonstrate that purified H3.3-G34R from isogenic HEK293 cells had an increased protein–protein interaction with HIST3H2A and HIST1H2AC [38], and Huang et al. [30] demonstrated that H3.3-G34V co-enriches with H3.3-wt. Another possible explanation is that H3K36me3 levels are increased in other oncogenes, such as MYCN, balancing the decreased levels in *cis* [33,39]. The changes in the distribution of H3K36me3 may also be mediated by H3.3-G34R binding and sequestering K9/K36 demethylases of the KDM4 family, enzymes responsible for the demethylation of H3K36me3 and H3K9me3 [33]. Indeed, the data demonstrated that G34R-mutation bearing cells exhibited an increased H3K36me3 and H3K9me3 signal in specific genes that are normally bound by KDM4 proteins [33]. Although this is one hypothesis for how H3FA-G34R/V mutations exert a dominant effect on oncogenesis, it is not as definitive as the mechanisms underpinning H3K27M mutations, which are known to affect both *cis* and *trans* histones by binding PRC2 [40]. 

H3K36me2/3 is thought to antagonize PRC2, the enzyme responsible for the methylation of H3K27, and in the absence of H3K36me3, PRC2 activity is thus increased [26]. As a result, H3F3A-G34R/V mutations decrease the SETD2-catalyzed formation of H3K36me3, which allows normal PRC2 to bind to H3, resulting in increased levels of H3K27me3 [26]. H3K27me3 is one of the hallmarks of the epigenetic silencing of genes and is found in tightly packed “inactive” heterochromatin [41]. There have been contradictory results regarding whether H3-G34 mutations allow for preserved NSD2 activity and H3K36me2 levels, but there is an increased proportion of H3K36me1 in H3-G34 mutants, and there is still at least some H3K36me2 [20,26,28,34,36]. Regardless, the decrease in H3K36me3 in *cis* is thought to be involved in the increase in H3K27me3 in *cis*, and these two alterations combine to contribute to gene silencing by inducing a heterochromatin formation and inhibiting the accessibility of transcription initiation proteins. However, despite the observation of decreased H3K36me3 and increased H3K27me3 in H3.3-G34R/V mutants [26,30], BioID experiments suggest that the degree of interaction between PRC2 and H3.3-G34R is decreased overall [34]. This could possibly be indicative of an increased efficiency of the PRC2-catalyzed trimethylation of H3K27 on the H3.3-G34R mutant histone [34] or may involve a complex interplay with Rpp29, as Rpp29-ko resulted in decreased H3.3K27me3 and increased the number of unmodified histones from multiple different histone families [37].

H3K36me3 is recognized by ZMYND11, a protein involved in transcription elongation and alternative splicing [42]. Given the effects of H3.3-G34R/V mutations on the altered distribution of H3K36me3 mediated by SETD2 inhibition [29] and co-enrichment with H3.3-wt [30], H3.3-G34R/V mutations alter ZMYND11 binding affinity [43]. Bressan et al. demonstrated that H3.3-G34R was enriched at active promoters and gene bodies, especially at intragenic CpG islands, and the knockout of G34R resulted in the downregulation of the genes that were previously enriched with H3.3-G34R [42]. Funato et al. built upon this by demonstrating that the last intron of NOTCH2NL had decreased amounts of H3K36me3 and a decreased binding of ZMYND11, which resulted in a decreased transcription of the intron and increased levels of properly transcribed functional NOTCH2NL mRNA [44]. The substitution of the H3.3-G34R mutation with an H3.3-K36R mutation, which cannot be methylated at the 36th amino acid, also resulted in these changes to NOTCH2NL expression [44].

The examination of H3K36ac patterns in H3.3-G34 mutant fission yeast revealed a decrease in the monoacetylation of H3K36 in *cis* on the mutant H3.3-G34R histone but not H3.3-G34V [36,45]. Gcn5 activity was markedly decreased on H3.3-G34R histones but only slowed on H3.3-G34V histones [45]. The differential expression of H3K36ac, which is typically enriched in the promoter of active genes [46,47], further demonstrates the diverse epigenetic effects of the oncohistone H3.3-G34R/V mutations. Siddaway et al. additionally demonstrated that the H3.3-G34R mutation resulted in lost interactions with a number of histone-modifying proteins, including methylases, demethylases, acetylases, and deacetylases, suggesting that the entire landscape of the epigenetic modifications and their effects upon cellular processes remain to be fully understood [34]. 

### 3.4. Histone H3.3-G34R/V Mutations Affect DNA by Altering Methylation Patterns, Repair Mechanisms, and Genetic Stability

H3.3-G34R/V mutants have been well described as exhibiting unique DNA hypomethylation patterns [8,43,48,49,50]. Siddaway et al. demonstrated that over 10,000 regions are differentially methylated when comparing H3.3-wt gliomas and H3.3-G34R gliomas, and over 88% of these were relatively hypomethylated in the H3.3-G34R gliomas [34]. The mechanisms underlying the hypomethylation likely directly involve the binding of DNA methyltransferases to chromatin; mutant histones H3.3-G34R/V demonstrate decreased interactions with DNA methyltransferase1 (DNMT) and DNMT3B [43], and H3.3-G34R in particular has been shown to have decreased interactions with DNMT3A as DNMT3A recognizes and binds H3K36me2 [34,43]. DNA methylation and H3K27me3 are antagonistic and do not co-occur, and since the H3.3-G34R mutant histone results in changes to histone distribution with resultant changes to PTM distribution, genes that are normally under the control of H3K27me3 may instead become regulated by DNMT3A in H3.3-G34 mutant gliomas [43]. The redistribution of DNMT3A could be a possible explanation for the high rate of MGMT methylation [4,51] in the setting of diffuse DNA hypomethylation.

One consequence of the aforementioned decreased H3K36me3 is the reduction of MutSα affinity, a DNA mismatch repair (MMR) enzyme, as the Proline–Tryptophan–Proline–Tryptophan (PWWP) domain of MutSα normally interacts with H3K36me3 [28]. Under normal conditions, this acts to recruit MutSα to active chromatin in order to repair mismatches, but in mutant-harboring cells, this mechanism is impaired. Fang et al. additionally demonstrated that H3G34R/V mutations result in a mutator phenotype [28]. Although Fang et al. did not note a defect in the homologous recombination (HR) repair of DNA [28], other studies concluded that the defective HR repair of DNA resulted in the genomic instability of H3.3-G34R cells [32,36,45], and the models of H3.3-G34R mutant gliomas created by Haase et al. also demonstrated inefficient nonhomologous end-joining repair (NHER) [32]. This quantitatively translated to decreased levels of DNA repair and cell cycle proteins as well as rates of DNA repair [32]. Their results concordantly demonstrated genomic instability in H3.3-G34R mutants [32].

The H3.3-G34R/V mutants may exhibit different phenotypes, although studies regarding this are conflicting. Lowe et al. noted that H3G34V fission yeast were still able to carry out HR repair and did not demonstrate the same degree of genomic instability as H3G34R [45]. Additionally, H3.3-G34R mutant cells were sensitive to replicative stress (hydroxyurea to decrease cellular deoxyribonucleotides, methyl methanesulfonate to alkylate DNA, and camptothecin to inhibit topoisomerase I) while H3.3-G34V cells were not. Their results suggested that G34V mutants were sensitive to ionizing radiation while G34R mutants were not [45], but Haase et al. demonstrated that H3.3-G34R mutant gliomas were more sensitive to radiation than H3.3-wt gliomas [32]. Lemon et al. demonstrated that both H3.3-G34R and H3.3-G34V mutant *Saccharomyces cerevisiae* yeast were sensitive to formamide, which inhibits RNA metabolism, and hydroxyurea [31]. Genetic instability is supported by the molecular analysis of resected H3.3-G34R/V mutant gliomas [52].

### 3.5. TP53, ATRX, and PDGFRA Are Heavily Implicated in Tumorigenesis of H3.3-G34R/V Mutant Gliomas

TP53 is a well-known tumor suppressor gene [53] that is frequently mutated in H3.3-G34R/V mutant gliomas [4,54]. TP53 mutations were incorporated into many of the models generated to study H3.3-G34R/V mutant gliomas [48,55,56,57,58]. Its specific role in the gliomagenesis of pediatric DHG is not understood, but p53 is a well-known tumor suppressor that is commonly mutated in cancers. In H3.3-G34R/V mutant gliomas, TP53 mutations lead to nonfunctional mutant p53 that exhibits nuclear accumulation. The detection of either can contribute to a diagnosis, but TP53 mutation and the resultant mutant p53 protein are sensitive and not specific for H3.3-G34R/V mutant gliomas. Although a selection advantage is hypothesized to contribute to the acquisition of TP53 mutations in certain gliomas, definitive evidence is lacking [59].

ATRX has been observed to be frequently mutated (typically in at least 80% of patients in case series but up to 100% in some) in H3.3-G34R/V mutant gliomas [50,51,52,60,61,62,63,64,65,66,67,68,69,70,71,72,73,74,75,76,77,78,79,80,81,82,83,84,85]. ATRX is found on the X chromosome and cooperates with Daxx to form a complex that allows for histone H3.3 deposition at telomeres. ATRX may contribute to gliomagenesis by promoting tumor cell immortality through the alternative lengthening of telomeres (ALT) [80]. The loss of KDM4B is also thought to contribute to ALT [55], and Voon et al. demonstrated that H3.3-G34R may bind and inhibit KDM4B [33]. Interestingly, ATRX-ko mice in an H3.3-G34R mutant glioma model designed by Abdallah et al. had an increased tumor latency, lower rates of ependymal cell differentiation, and the upregulation of HOX genes [56]. When combined with the H3.3-G34R mutation, ATRX-ko resulted in the upregulation of neuronal markers. Thus, at least when combined with the H3.3-G34R mutant histone, ATRX may result in altered transcriptomics. Although studies have not found a statistical difference in incidence related to sex, the largest systematic reviews do note that there are more male patients than female (approximately 1.5:1 ratio) [4,54], which could be related to the location of the ATRX locus on the X chromosome as males only have one X chromosome. The penetrance of the H3F3A-G34R/V mutations is not known, but it is possible that the glioma phenotype is not 100% penetrant. 

PDGFRA is also mutated or overexpressed in a large proportion of H3.3-G34R/V mutant gliomas [51,52,72,83,85] and is commonly utilized in the generation of H3.3-G34R/V mutant glioma models [32,42,48,57,58]. The downstream signaling of PDGFRA increases cell survival and proliferation, and overexpression in gliomas may be a key tumorigenic step [86]. While a number of studies only focused on whether PDGFRA mutations or focal amplification were present, Carvalho et al. noted that a 4q12 rearrangement resulted in a PDGFRA mutation that was activating and tumorigenic [87]. Chen et al. additionally demonstrated in a mouse model that PDGFRA amplification was relatively uncommon but that PDGFRA overexpression was driven by changes in chromatin conformation such that PDGFRA was able to co-opt *cis* regulatory elements such as the GSX2 enhancer hs687 [57]. This is likely not relevant in human patients as GSX2 and PDGFRA are on different chromosomes in humans, but other genes could contribute to the overexpression of PDGFRA. They also interestingly noted that PDGFRA was present at a higher rate in recurrent H3.3-G34R/V mutant gliomas. They hypothesize that the H3.3-G34R/V mutation may become dispensable following the acquisition of PDGFRA overexpression and that PDGFRA activates downstream MAP/ERK signaling, increasing the clonogenicity and expansion of the astrocytic compartment of the tumor (at the expense of the neuronal components) [57]. Other upstream effectors of the MAP pathway such as other receptor tyrosine kinases have also been implicated in the molecular analysis of tumor samples [2].

Various PDGFRA mutations have been identified, including in the extracellular immunoglobulin domains, the transmembrane domain, and the tyrosine kinase domain that lies in the intracellular compartment [67]. Two important PDGFRA mutations were utilized by McNicholas et al. to generate a syngeneic transplantable model: PDGFRA-D842V and PDGFRA-C235Y [58]. PDGFRA was indispensable to tumorigenesis, and without the H3.3-G34R mutation, the introduction of the PDGFRA mutations was highly lethal. The PDGFRA-D842V mutation resulted in 100% penetrance, while PDGFRA-C235Y and PDGFRA amplification was only 55% penetrant. Interestingly, the cells bearing the PDGFRA-C235Y mutation were not sensitive to avapritinib, a PDGFRA inhibitor, but they were sensitive to infigratinib, an FGFR inhibitor [58]. Lucas et al. also noted that the D842V mutation, which occurs in exon 18, results in resistance to imatinib, which theoretically would inhibit the tyrosine kinase activity of the intracellular domain of PDGFRA [67].

However, as PDGFRA is not mutated in 100% of H3.3-G34R/V mutant gliomas, perhaps there are different ways that the mutant cells can become gliomas, similar to how PDGFRA reflects an oligodendroglial differentiated type of diffuse intrinsic pontine glioma (DIPG) in contrast to a mesenchymal type [88]. This implies the existence of H3.3-G34R/V mutant glioma subtypes or suggests that while the H3.3-G34R/V mutation is oncogenic, other mutations may guide a subsequent diagnosis, as is seen in the IDH mutant astrocytoma and oligodendroglioma [89]. Perhaps in support of this is the differential frequency of PDGFRA and CCND2 amplifications in tumors identified as GBM versus a primitive neuroectodermal tumor (PNET) in a series reported by Korshunov et al. [77]. Additionally, in one analysis, the only H3.3-G34R/V mutant glioma with both TP53-wt and ATRX-wt harbored a PDGFRA mutation [85] (although this mutant also harbored a TERT promoter mutation, which is not thought to be associated in the gliomagenesis of H3.3-G34R/V mutants). Further research utilizing mutated human or mouse forebrain neuroprogenitor cells (discussed below) in an immunocompetent environment may shed light on how the H3.3-G34R/V oncohistone leads to the acquisition of various key oncogenes such as PDGFRA. 

### 3.6. Forebrain Neuronal Identity Is a Critical Component of Tumorigenesis of H3.3-G34R/V Mutant Gliomas

Research suggests that H3.3-G34R/V mutant gliomas originate from embryonic neuronal precursors that arise in the ganglionic eminence [44,57]. Based on gene signatures, it is suggested that these cells would have been fated to become inhibitory interneurons. GSCX2 and DLX1/2, key transcription factors for specification to an interneuron fate and the inhibition of specification to an oligodendrocyte fate, are preserved in H3.3-G34R/V mutant gliomas [57]. The loss of Olig2, an oligodendrocyte marker, is a well-described histopathologic finding of H3.3-G34R/V mutant gliomas [68,90], although focal positivity in co-occurring reactive glial cells has been described [74]. FOXG1, a transcription factor that is commonly expressed in H3.3-G34R/V mutant gliomas [91] and considered the “master regulator of forebrain identity”, was also demonstrated to be essential to H3.3-G34R gliomagenesis. There is an inverse correlation between FOXG1 and Olig2 levels, which supports the divergent differentiation of these glioma cells [91]. The transition away from an oligodendrocyte to an interneuron fate may explain why Huang et al. described the upregulation of genes associated with cellular neuron projection morphogenesis and neuronal differentiation [30]. However, the expression of forebrain markers was not altered by the introduction of H3.3-G34R, PDGFRA amplification, and TP53-ko mutations into forebrain neural stem cells (NSCs), suggesting that these mutations do not transform cells to resemble forebrain NSCs but that these are the cell-of-origin [42].

FOXG1 likely contributes to clonogenicity in addition to determining forebrain identity. The deletion of FOXG1 resulted in the upregulation of p21, which is encoded for by CDKN1A and plays a role in cell senescence [42]. The effects of p21 appear to be mediated by the inhibition of cyclin-dependent kinases and arrest of the cell cycle. This pathway is activated by telomeric depletion and/or DNA damage [92]. Thus, cells with FOXG1 deletions accordingly demonstrated a decreased proliferation [44]. Hindbrain NSCs transfected with both H3.3-G34R and TP53-ko mutations (with or without PDGFRA amplification mutations) resulted in increased CDKN1A mRNA and cellular levels of p21. Additionally, these mutations resulted in a cytostatic effect with a significant reduction of the colony count. This effect was not observed when hindbrain NSCs were transfected with the H3.3-G34R mutation alone [42]. Thus, the H3.3-G34R and TP53-ko mutations likely work in combination with FOXG1 to reduce CDKN1A and p21 expression in H3.3-G34R/V mutant glioma cells.

The ganglionic eminence is the source of embryonic neuronal stem cells that ultimately migrate to various lobes of the forebrain [93]. Bressan et al. demonstrated that there is the upregulation of forebrain-specific transcription factors DMRTA2, EMX2, NR2F1, and HIVEP2 in H3.3-G34R mutant cells [42]. Interestingly, when they transfected hindbrain fetal NSCs with the H3.3-G34R mutation, PDGFRA amplification, and a TP53-ko mutation, the stem cells became cytostatic. The importance of regional identity is reinforced by the observation that the H3.3-G34R/V mutations and H3K27M mutations have been mutually exclusive in patients with high-grade gliomas [2,8]. This supports the hypothesis of the differing cells-of-origin of the two diseases. Although there have been clinical case reports of co-occurring H3G34R and H3K27M mutations, it is important to note that in both cases, the G34R mutation occurred on H3.1 [63,94], which is not a constitutively expressed histone variant, unlike H3.3.

Funato et al. also noted differences in behavior between forebrain and hindbrain neural progenitor cells (NPC) as forebrain NPCs with ATRX-ko, TP53-ko, and H3.3-G34R mutations formed rosettes (which are normally only transiently formed during differentiation) and formed tumors in vivo when combined with N-Myc (discussed below) [44]. The forebrain and hindbrain NPCs additionally clustered separately when RNA sequencing was analyzed, with forebrain NPCs harboring the aforementioned mutants clustering patient-derived H3.3-G34R mutant glioma cells [44]. Their results also suggest an arrested state in differentiation where the cells act as a stem cell population. One example is the demonstration of ASLCL1 downregulation, a transcription factor essential for neuronal differentiation. 

On another note, the destination of ganglionic eminence NSCs is thought to be determined with embryonic day 13.5 of life [95], which questions the generalizability of models produced in fetal and postnatal organisms/cells. McNicholas et al. created a syngeneic engraftable model utilizing in utero electroporation at embryonic day 12.5 of life but noted that this process was not successful in vitro and that an in vivo immunocompetent environment is necessary [58]. 

### 3.7. Other Possible Key Proteins and Pathways Involved in Gliomagenesis

In a comparison of KNS42 cells, a cell line derived from a HGG patient harboring G3.3-G34V, versus SF188, a cell line derived from a glioblastoma patient with H3.3-wt, Bjerke et al. reported a marked increase in the enrichment of H3K36me3 at the MYCN locus and resultant expression [39]. They did not find focal gene amplification, although the patient in the series by Trabelsi et al. did exhibit MYCN amplification [96]. Rpp29-ko was associated with an increased MYCN expression [37], and N-Myc overexpression was utilized by Funato et al. to create a graftable G3.3-G34R mutant glioma model harboring ATRX-ko and TP53-ko mutations [44]. Mackay et al. additionally noted that whole-arm 4q deletions were present in some H3.3-G34R/V mutant gliomas, which knocked out FBXW7, an oncogene that regulates the expression of MYC/MYCN by targeting it for proteasomal degradation [2]. Thus, MYCN may be a key protein in gliomagenesis, and its overexpression could occur through a number of different pathways.

The Notch pathway and its downstream products are implicated in H3.3-G34R/V gliomagenesis, as Funato et al. demonstrated the upregulation of HES1, HEYL, and NOTCH2NL [44]. The overexpression of NOTCH2NL was found to be partially due to the alteration of ZMYND11 binding. Specifically, the last intron of NOTCH2NL had decreased levels of H3K36me3, which resulted in a decreased ZMYND11 binding and resultant loss of intron retention, leading to increased functionally spliced NOTCH2NL mRNA [44]. This provides one possible mechanism by which gene expression can be altered by H3.3-G34R/V gliomas: alternative splicing. The authors did note that the NOTCH2NL locus was amplified in 44% of H3.3-G34R mutant gliomas analyzed; however, the upregulation of NOTCH2NL could be due to two separate mechanisms [44]. Additionally, Siddaway et al. demonstrated that H3.3-G34R had a lower affinity for SPEN, a gene involved in the repression of NOTCH [34]. Lowe et al. demonstrated that Sgo2, a protein involved in mitosis that can silence genes, localizes to subtelomeric domains in both H3K36ac-deficient cells and in fission yeast with the H3.3-G34R mutation, forming “knobs” and silencing the genes within the subtelomeric domains [45]. While this is completely conjecture, this mechanism could hypothetically alter the balance of Sgo2 binding and change the expression of genes located in loci outside of the subtelomeric domains, such as NOTCH2NL, which is located far from the subtelomeric domain of chromosome 1q [97,98]. 

The tumor microenvironment is a hot topic in oncology, and gliomas add a layer of complexity to this given the presence of the blood–brain barrier [99]. Jiao et al. demonstrated that RACK7 (also known as ZMYND8) binds to the H3.3-G34R mutant histone and decreases the transcription of CIITA, which is known to upregulate MHC II genes, their chaperones, and CD74 [100]. RACK7 also downregulated the expression of junction proteins and vesicle-related genes. The effects of differential MHC II expression are unclear and controversial; however, some studies report a poorer prognosis with MHC II expression [101,102]. Zagzag et al. demonstrated that the downregulation of MHC II could contribute to the infiltrative nature of gliomas [103]. In H3.3-G34R/V mutant gliomas, this could conceivably be a basis for the relatively low peritumoral edema seen on imaging. 

Other immune pathways appear to be dysregulated in H3.3-G34R/V mutant gliomas as well. LIF, a cytokine that is part of the IL-6 family, contributes to the phosphorylation of Stat3, which plays a role in the maintenance of murine embryonic stem cell populations [104]. LIF can be secreted by cells and upregulate downstream pathways via autocrine and paracrine signaling mechanisms [48]. Sweha et al. demonstrated in an H3.3-G34R mutant mouse NSC model that enriched H3K36me3 is enriched at LIF and JAK/STAT signaling genes [48]. They additionally noted that the LIF promoter was hypomethylated and enriched with H3K27ac and H3K4me3. The chromatin at the LIF locus exhibited a conformation consistent with active transcription, LIF mRNA was increased, and phosphorylated Stat3 (pStat3) was increased, which supported the upregulation of the LIF and the downstream JAK/STAT pathway in H3.3-G34R/V mutant gliomas. Utilizing shRNA directed at LIF, they demonstrated that the inhibition of this pathway resulted in changes of the expression of genes involved in cell differentiation and motility, apoptosis and cell death, and neuronal differentiation. They additionally utilized Stattic and WP1066, small-molecule inhibitors of Stat3, and demonstrated the growth inhibition and cellular toxicity of G34R/V mutant glioma cells. Both Stattic and LIF shRNA reduced tumor volumes, and WP1066 resulted in an increased survival [48]. They additionally hypothesized that radiation therapy in combination with WP1066 may be more effective than radiation therapy alone.

Khazaei et al. demonstrated that H3.3-G34R/V mutations in mice resulted in differing phenotypes, with H3.3-G34R mice developing ataxia and neuromuscular defects [43]. H3.3-G34W (associated with giant cell tumors of the bone [17]) resulted in defects related to mesenchymal tissues, and H3.3-G34V resulted in phenotypes that lay in the middle of the spectrum between H3.3-G34R and H3.3-G34W [43]. The H3.3-G34R mice demonstrated a loss of layer 5 and layer 6 glutaminergic neurons, which was determined to be due to the upregulation of complement genes and resultant inflammation mediated by microglia. DNA hypomethylation, as a result of the H3.3-G34R mutation, led to the downregulation of NPTX2, a neuronal gene, and resulted in continued complement signaling, which is thought to normally serve a key role in recruiting the supporting microglia and astrocytes to neurons during early development [43]. The inappropriate expression of the complement cascade contributed to neuronal loss.

The non-nuclear expression of H3.3-G34R/V may contribute to tumorigenesis. Siddaway et al. demonstrated that the H3.3-G34R mutation resulted in the differential expression of genes involved in cellular metabolism. They also demonstrated that some of the proteins that interact with H3.3-G34R localized to mitochondria. While the majority of H3.3-G34R localizes to the nucleus, the simultaneous staining of H3.3-G34R and mitochondria revealed a small degree of colocalization, suggesting that H3.3-G34R may regulate mitochondrial metabolism [34]. They also reported the upregulation of the tricarboxylic acid cycle, arginine synthesis, and metabolism of cysteine and methionine. To a lesser extent, they observed the upregulation of pyruvate, alanine, aspartate, and glutamate metabolism [34]. Although the significance of this is unclear, they additionally noted differential concentrations of various citric acid cycle metabolites; levels of most intermediates were increased with the exceptions of decreased early intermediates linking glycolysis to oxidative metabolism (acetyl-CoA and citrate) as well as succinate. Additional research is needed to provide insight into the effects of the H3.3-G34R/V oncohistones on mitochondrial metabolism and the potential effects of altered mitochondrial metabolism on epigenetics.

Other genes are differentially expressed in H3.3-G34R/V mutant cells as well, but their roles in oncogenesis have not yet been elucidated. HOXA2, HOXA3, HOXA5, HOXA7, COL5A1, COL6A2, KHDC8A, PDGFD, and PGM5 are only some of the differentially expressed genes that were identified in a single study [56]. Additionally reported is the possibility of fusion genes such as CSGALNACT2:RET and DHX57:TMEM178:MAP4K3 [87]. Lim et al. introduced the H3.3-G34R mutation to HEK293 and discovered that this mutation resulted in 20 unique interactions with proteins that were not observed in H3.3-wt, H3.3-G34W, or H3.3-K27M [38]. There is varying data on the frequency of co-occurring CDKN2A/2B mutations [62,64,67,75]. The cyclin D-CDK4 pathway involving Rb may also be implicated [75], and EGFR has been associated with a poorer prognosis [77]. Chromosomal translocations are also frequently observed [105]. Siddaway et al. also reported that H3.3-G34R increases cryptic transcription [34]. Given the numerous other mutations and altered epigenomes observed in H3.3-G34R/V mutant gliomas, there are likely even more derangements of cellular processes involved in gliomagenesis. However, additional studies are needed to determine which mutations are necessary and sufficient for gliomagenesis. The results of our review of studies that created models to investigate the molecular pathways involved in H3.3-G34R/V gliomagenesis can be found in Table S1.

### 3.8. Baseline Information and Clinical Presentation of Patients with H3.3-G34R/V Mutant Gliomas

Sixty-two studies reported information regarding patient cases of H3.3-G34R/V mutant gliomas (not including patient-derived glioma cells), of which sixteen were case reports. The entire patient samples of seven of these studies were included in another study. The median number of H3.3-G34R/V mutant glioma patients in each study was four patients. The incidence of H3.3-G34R/V mutant gliomas peaked in adolescence with a median age at diagnosis between 15 and 17 years of age [4,54]. H3.3-G34R/V mutant gliomas made up approximately 5–15% of pediatric HGGs/GBMs [81,82,83,106,107]. When studies included adults with HGG or GBM, the prevalence of the H3.3-G34 mutant glioma subgroup was 1% [96] and 8.6% [8], respectively, although the selection criteria for adult patients in the study by Sturm et al. were not well delineated. When adults with IDH-wt anaplastic astrocytoma (WHO grade 3) and diffuse astrocytoma (WHO grade 2) were focused upon, H3.3-G34R mutant gliomas made up approximately 2% of the sample [50]. In studies that focused on patients older than 18 years of age, the median age of diagnosis was frequently around 24–25 years of age [50,66,68,74,108], supporting that onset is skewed toward adolescence and early adulthood, with incidence in later adulthood being rare. As mentioned before, the reviews mentioned that there was no statistically significant sex difference in incidence, although there were more males than females with H3.3-G34R/V mutant gliomas. Excluding studies whose cohorts were well described as being included in another study, there were 470 cases of H3.3-G34R/V mutant gliomas. Data regarding sex were available for 438 patients (198 females, 45%), and data regarding the specific H3.3-G34 variant were available for 444 patients (418 H3.3-G34R, 94%). 

A number of studies reported on clinical presentation and/or survival [66,67,68,69,70,71,72,73,74,75,108,109,110,111,112,113,114,115,116,117,118,119,120,121,122,123]. Survival is discussed below with treatment regimens in a separate section. Reasons for initial presentation were most frequently signs and symptoms of high intracranial pressure (headache, nausea, vomiting, blurred vision, or somnolence), focal neurologic deficits, and seizures. Metastasis was uncommon but did occur in four cases, with the most common patterns being osseous metastasis and a leptomeningeal spread [69,109,110,114]. 

### 3.9. Radiologic Findings Common to H3.3-G34R/V Mutant Gliomas

On magnetic resonance imaging (MRI), H3.3-G34R/V mutant gliomas tended to be T1 hypo- to isointense and T2 hyperintense, display mild edema relative to the size of the mass, exhibit at least some degree of diffusion restriction, have ill-defined margins, and demonstrate an infiltrative growth pattern [73,74,75,120,121,124,125,126,127,128,129]. All were supratentorial with a variable contrast enhancement, although a significant proportion of tumors demonstrated minimal contrast enhancement or none. Other frequently observed characteristics include areas of a hemorrhage, leptomeningeal contact, ependymal contact, and cystic or necrotic components. The frontal, parietal, and temporal lobes were the most common locations with the involvement of the occipital lobe to a lesser extent. The involvement of the corpus callosum and extension from a hemispheric location to the basal ganglia were not uncommon. Multifocality and/or gliomatosis were also reported. The lobar location of pediatric-type glioma was associated with an older age at diagnosis, compared to brainstem locations, which are associated with a younger age at diagnosis, further supporting that these are distinct clinical entities.

Computed tomography (CT) is not the imaging modality of choice for the characterization of brain tumors but is commonly the first imaging acquired, especially when patients present to an emergency department. For H3.3-G34R/V mutant gliomas, CT findings were relatively nonspecific. Tumors exhibited various degrees of density, and calcifications were present in some cases [120,128].

The findings on advanced imaging modalities were also relatively nonspecific with the reversal of Hunter’s angle on MRI spectroscopy being a common finding [73,120,121,128]. Lactate and/or lipid peaks were also frequently observed. Perfusion studies tended to demonstrate hyperperfusion [73,120,121,125,128], although hypoperfusion was occasionally seen. High uptake on 18F-fluoroethyl-L-tyrosine positron emission tomography (18F-FET-PET) was observed [126]. Overall, H3.3-G34R/V mutant gliomas may radiographically resemble either low-grade or high-grade gliomas, although patient age may help to narrow the differential diagnosis. The presence of leptomeningeal invasion or osseous metastases may also provide another clue for narrowing the differential, but tissue sampling and a molecular analysis remain essential to establish the diagnosis.

### 3.10. Histopathologic Analysis and Subsequent Diagnosis of H3.3-G34R/V Mutant Gliomas

H3.3-G34R/V mutant gliomas can display diverse histopathologic features. Generally, the tumors resemble embryonal tumors [71,72] or HGGs [77], and mixed glial and neuronal components are often present [60,63,76,112,130]. Mitoses are often present but not to the same degree as seen in other HGGs. Microvascular proliferation and necrosis are frequently observed, and Homer Wright rosettes can be seen as well. Neuronal features, ganglionic features, and even gemistocytes have been observed [63,84,112]. Positivity for glial fibrillary acidic protein (GFAP) is common, and synaptophysin positivity is more variable. The nuclear accumulation of p53 and abnormal Atrx expression on immunohistochemistry (IHC) is highly prevalent due to the frequency of TP53 and ATRX mutations co-occurring with H3.3-G34R/V. Nearly all tumors were negative for Olig2 in concordance with molecular studies indicating that the H3.3-G34R/V mutation results in a shift away from oligodendroglial differentiation [57], and Picart noted that Olig2 could be falsely reported as positive to the presence of reactive glial cells [74].

H3.3-G34R/V mutant gliomas have histopathologically been diagnosed as a number of other entities in the past including anaplastic pleomorphic xanthoastrocytoma [60], GBM, GBM with epithelioid features [112], small-cell GBM [81], gemistocytic astrocytoma [63], and PNET [71,72]. Of these, H3.3-G34R/V mutant gliomas exhibited a considerable overlap of histopathologic features with GBM and PNET. The heterogeneity of tumors previously classified as PNET was recognized, and PNET is no longer a diagnosis recognized by the WHO [89]. Korshunov et al. demonstrated that re-classifying 84 samples previously diagnosed as PNET by histopathologic features was not a feasible task [72]. Of 85 participants in the ACNS0332 trial, which included patients with newly diagnosed PNET and pineoblastoma, 8 patients were subsequently diagnosed with H3.3-G34R/V mutant gliomas [71].

The question remains of how to diagnosis H3.3-G34R/V mutant gliomas in a cost-effective and efficient manner. Next-generation sequencing is commonly employed in the diagnosis of gliomas given the diverse genetic landscape and resultant differences in treatments. Haque et al. developed an anti-H3.3-G34R and anti-H3.3-G34V antibody [131], and Huang et al. reported that H3.3-G34V mutant glioma cells were detected in the cerebrospinal fluid of a patient who had a DHG with thalamic extension [132]. Yoshimoto et al. also performed the IHC of various methylated H3 histones [76]. However, studies have demonstrated that utilizing IHC alone led to high rates of inaccuracy [65,133], and the feasibility of a cerebrospinal fluid analysis has not been studied on a larger scale. However, in the absence of a leptomeningeal spread, a cerebrospinal fluid analysis may involve a low yield. Trejo-Lopez suggested that H3.3-G34R/V testing can be reserved for patients younger than 50 years of age with tumors that exhibit primitive neuronal features or abnormal ATRX, p53, or Olig2 IHC results [68].

### 3.11. Treatment, Outcomes, and Factors Associated with Improved Prognosis in Patients with H3.3-G34R/V Mutant Gliomas

Treatments often included biopsy or resection followed by chemotherapy and/or radiation therapy [3,51,72,120,121,123,124,134], with a few studies having employed craniospinal radiation [3,72,114]. Hemispheric tumors are considered more resectable due to an easier accessibility and remoteness from deeper structures essential for basic survival functions, with one meta-analysis detailing adult patients with high-grade gliomas of all types undergoing gross total resection in nearly 50% of hemispheric tumors but only approximately 20% of midline gliomas [135]. It is important to note that a large portion of DHGs were subtotally resected given the size, deep infiltration, and multifocal nature (such as gliomatosis [134,136]) of the tumors. The most common chemotherapeutic agents utilized included temozolomide [3,119], vincristine [71,72], lomustine [63,74,120], cisplastin [3,51], bevacizumab [62,79,123,124], and procarbazine [63,74], although other drugs, such as methotrexate, etoposide, and carboplatin or thiotepa, carboplatin, and cyclophosphamide [72]; doxorubicin, mesna, and ifosfamide [69]; irinotecan [62]; nimustine [76]; isotretinoin [71]; and dianhydrodulcitol [51], were also utilized. A standard chemotherapeutic regimen does not exist, and regimens are extrapolated from other studies. Deciding upon a standard of care is difficult due to the heterogeneity of the available studies. For example, while the multicenter HERBY phase II trial demonstrated that bevacizumab was detrimental, the single institution series reported by Crotty et al. demonstrated that temozolomide in combination with bevacizumab (with the addition of irinotecan) was tolerable and non-inferior [62]. Studies that specify the particular treatment utilized, including surgery, adjuvant radiation therapy, and chemotherapeutic regimens, can be found in Table 1. The median survival was in the range of 14–17 months in the largest systematic reviews [4,54], and in the reviewed case series and cohorts containing at least 10 patients, the median survival was as low as 12 months [74,75] and as high as 30 months [119].

Due to study heterogeneity and a lack of complete follow up, conducting a meta-analysis of H3.3-G34R/V mutant glioma patients with all of the studies with patient-specific data is not feasible. Six studies reported on a multivariable analysis investigating factors associated with survival. Two of these studies reported on the same patient sample [122,124]. Rodriguez Gutierrez et al. conducted a radiologic review of the HERBY phase II trial [124] and reported an improved survival with gross total resection or near total resection, but Grill et al.’s multivariable analysis did not demonstrate a statistically significant improvement in survival although the Kaplan–Meier curves were suggestive of an effect with gross/near total resection [122]. Rodriguez Gutierrez also noted a worsened survival with a leptomeningeal spread, but neither article reporting on the results of the HERBY phase II trial conducted a multivariable analysis solely on the H3.3-G34R/V mutant group. MGMT methylation and gross total resection were associated with an improved survival [3,66]. Ill-defined margins, TP53 mutation, and oncogene amplification (specifically PDGFRA, EGFR, CDK4, MDM2, CDK6, CCND2, or MYCN) were associated with a worsened survival [3,66,120]. The analysis of the ACNS0332 trial noted that supratentorial high-grade glioma (which included H3.3-G34R/V but did not separate it as subgroups for the survival analysis) portended a poorer prognosis than a supratentorial embryonal tumor or pineoblastoma [71]. Overall, these results suggest that there remains much room for the improvement of outcomes in patients with H3.3-G34R/V mutant gliomas and that this population is understudied.

### 3.12. Possible Therapeutics and Clinical Trials (Future Directions)

Studies in both in vitro and animal models demonstrated that a number of different agents can inhibit H3.3-G34R/V mutant glioma growth or lead to cell death. While some studied treatments targeted at the epigenetic modifications induced by the oncohistone, others focused on other dysregulated cellular pathways. Siddaway et al. demonstrated that OTS186935, an inhibitor of SUV39H2, and chaetocin, an inhibitor of SUV39H1/2 and EHMT1/2, had a lower IC50 in H3.3-G34R mutant cell lines compared to H3.3-wt cell lines [34]. These enzymes catalyze the trimethylation of H3K9, suggesting that this epigenetic modification may be a possible target for the treatment of H3.3-G34R/V mutant gliomas. Vorinostat, a histone deacetylase inhibitor, was also shown to be effective in combination with poly-ADP ribose polymerase inhibitors (PARPi) [137].

As all of the contributory pathways to H3.3-G34R/V mutant gliomas have yet to be elucidated, there may be many other possible treatments yet to be discovered. Sweha et al. demonstrated the efficacy of Stattic and WP1066, small molecule inhibitors of Stat3. Stattic and short hairpin RNA against LIF decreased the tumor volume in mice that were xenografted with KNS42 (patient-derived H3.3-G34V mutant glioma) cells [48]. WP1066 resulted in a survival benefit and, in combination with radiation therapy, was more effective than radiation therapy alone. McNicholas et al. demonstrated that cells derived from a H3.3-G34R mutant glioma model containing the PDGFRA-C235Y mutation were not sensitive to the PDGFRA inhibitor avapritinib but were highly sensitive to the FGFR inhibitor infigratinib [58]. Lucas et al. additionally noted that PDGFRA-D842V was associated with insensitivity to imatinib [67]. While veliparib did not demonstrate efficacy in the treatment of pediatric HGG [138], other PARPis have shown promise in both in vitro and animal models [32,137]. Both olaparib and niraparib were effective in combination with vorinostat [137], and pamiparib was demonstrated to be more potent than veliparib [32]. Additionally, veliparib did not increase the efficacy of radiation therapy [34]. However, it is important to note that veliparib has a high blood–brain barrier permeability, and drug delivery across the blood–brain barrier remains a challenge.

As H3.3-G34R/V mutant glioma cells were demonstrated to possibly be sensitive to replicative stress, it would be reasonable to conclude that processes that mimic a state of cellular stress may inhibit tumor growth. eIF2α is a subunit of eIF2, which plays a key role in protein translation [137]. It is phosphorylated to P-eIF2α during cellular stress. Salubrinal and raphin-1, inhibitors of P-eIF2α dephosphorylation (resulting in increased levels of P-eIF2α), decreased the survival of KNS42 cells, with raphin-1 being more potent. Salubrinal and raphin-1 led to an increased ratio of P-eIF2Bϵ/eIF2Bϵ, which suggests that they may also inhibit the ERK-1/2 pathway. Both inhibitors demonstrated a synergistic effect against the H3.3-G34V mutant cells when combined with olaparib. Raphin-1 and niraparib in combination increased the number of double-stranded DNA breaks [137]. Other cellular stressors demonstrated some promising results. Silencing RNA against MAP4K3, which is involved in mTOR signaling, and silencing RNA against cellular metabolism, was effective at reducing KNS42 cell viability [87]; AZD7762, a checkpoint kinase inhibitor that can impair DNA repair, also demonstrated promising results [32]. 

Immunotherapy may also be a potential therapeutic option for H3.3-G34R/V mutant gliomas. Haase et al. subjected an H3.3-G34R mutant glioma model to ionizing radiation and observed the release of interferon-beta in response to ionizing radiation [32]. This process was dependent on the cGAS/STING pathway, and the administration of diABZI, a STING agonist, resulted in a greater long-term survival in mice grafted with H.3-3G34R mutant glioma cells. On the other hand, knocking out CD8 abrogated any survival benefit that would have been gained with treatment. Additionally, H151, a STING antagonist, eliminated the efficacy of the combination treatment of pamiparib and radiation therapy, resulting in an even poorer survival than untreated mice with H3.3-G34R mutant glioma [32]. Interestingly, Haase et al. noted that long-term survivor mice had decreased rates of tumor formation upon subsequent inoculation, implying the role of adaptive immune memory. This suggests that H3.3-G34R/V mutant glioma vaccines or chimeric antigen receptor (CAR) T-cells are a possible avenue of research.

Vanan et al. discussed the possibility of treatments involving the MYCN pathway but noted that the direct inhibition of MYCN is not possible due to the protein being non-enzymatic and lacking targetable domains [139]. Suggested therapies include a reduction of the MYCN copy number (which would theoretically only be applicable in a small subset of tumors) [140] or drugs that inhibit proteins that increase MYCN levels such as Aurora-A kinase inhibitors (MK5108, MLN8054, and MLN8237), checkpoint kinase 1 inhibitors (SB21807 and TCS2312), bromodomain and extraterminal domain inhibitors (JQ-1, I-BET151, and I-BET762), the non-receptor tyrosine kinase (NRTK) 2 inhibitor lestaurtinib, and the pan-NTRK inhibitor AZ64 [139]. 

There is limited clinical trial data for H3.3-G34R/V mutants. The HERBY phase II trial included H3.3-G34R/V mutants, but these patients made up 7/121 (5.8%) of the sample [122]. Additionally, Mackay et al. reported that the H3.3-G34R/V mutant glioma patients in the HERBY II trial demonstrated a trend toward a decreased survival [141]. NCT04334863, which aimed to investigate the safety of WP1066 (previously mentioned in this review) in patients with a progressive or recurrent malignant brain tumor (which could include H3.3-G34R/V mutant gliomas), primary spinal tumors, or H3K27M mutant gliomas, was completed in February 2023. Other clinical trials are underway, which can also include H3.3-G34R/V mutant gliomas, such as NCT02977780, NCT04185038, and NCT04908176, but given the low prevalence of H3.3-G34R/V mutant gliomas, a subgroup analysis of these patients will likely not be a feasible task. There is currently one clinical trial planned that will investigate patients specifically with the progression or recurrence of H3.3-G34R/V mutant gliomas; NCT05457959 plans to investigate the efficacy of peptide-pulsed dendritic cell vaccination with the monoclonal antibodies nivolumab (anti-PD-1) and ipilimumab (anti-CTLA-4). This study may be a step forward for H3.3-G34R/V-mutant-glioma patients as the first clinical trial targeted at this particular disease. However, of the other drugs and RNA therapies mentioned, only veliparib and possibly WP1066 have been assessed in patients with H3.3-G34R/V mutant gliomas. There is clearly a paucity of high-quality data that can be utilized to provide a scaffold for treatment algorithms of H3.3-G34R/V mutant gliomas. This is limited in part by the rarity of this tumor subtype, which leads to relevant studies being underpowered.

## 4. Conclusions

The mutant oncohistone H3.3-G34R/V results in pediatric-type diffuse hemispheric glioma, which displays characteristic DNA methylation and histone alteration patterns. Although the mutation was first described in 2012, official WHO codification occurred in 2021. This review summarizes the relevant pathways that contribute to gliomagenesis, including the disruption of histone modifications with resultant changes in transcription, alternate splicing, the alternative lengthening of telomeres, dysfunctional DNA repair, genomic instability, and immune dysregulation. Given advances in high-throughput sequencing, there is a wealth of information available regarding the varying mutations, chromosomal abnormalities, gene copy number variation, transcriptomes, interactomes, and epigenomes associated with H3.3-G34R/V mutant gliomas. However, additional research is necessary to elucidate the exact processes by which this oncohistone leads to the development of diffuse hemispheric glioma, and there is a crucial need for investigational drug studies to improve the currently dismal patient outcomes.

## Figures and Tables

**Table 1:** Information regarding studies with newly reported cases of H3.3-G34R/V mutant gliomas. Age inclusion criteria are denoted if they were specified in the articles.

Study	Inclusion Age	Total Patients	Median Age (Years) ^a^	Number of Males/Females ^a^	Number of H3.3-G34R/V Mutants ^a^	Median EFS or PFS ^a,b,c,d,e^ (Months)	Median OS ^c,d,e^ (Months)	Treatments
Korshunov A et al. (2015) [119]	1–18 years	24	14	17/7	24/0	N/A	~30	24: Surgery (GTR or STR) followed by RT + TMZ. Recurrent tumors treated with polychemotherapy (not specified)
Hummel TR et al. (2015) [123]	3–30 years	1	22	0/1	1/0	19.6	22.9	1: GTR followed by RT + TMZ + BEV
Broniscer A et al. (2016) [134]	<22 years	4	12.2	3/1	4/0	N/A	11.4	4: Biopsy followed by RT + chemotherapy (not specified)
Korshunov A et al. (2016) [3]	All	81	19	42/32	77/4	9	22	48: Surgery (not specified) followed by RT + TMZ 12: Surgery (not specified) followed by combinations of craniospinal RT + polychemotherapy (various combinations of vincristine, lomustine, and cisplatin)
Salloum R et al. (2017) [79]	N/A	1	12	1/0	0/1	7.1	N/A	1: GTR followed by RT + TMZ and TMZ + BEV
Yoshimoto K et al. (2017) [76]	All	4	10.5	2/2	4/0	N/A	15.5	2: STR followed by RT + TMZ 1: STR followed by RT + cisplastin + vincristine 1: Biopsy followed by RT + nimustine
Hwang EI et al. (2018) [71]	3–22 years	8	14.6	5/3	N/A	14.7	23.4	1: Biopsy followed by RT + vincristine + carboplatin 1: STR followed by RT + vincristine + carboplatin 1: GTR followed by RT + vincristine + carboplatin 1: GTR followed by RT + vincristine + isotretinoin 4: GTR followed by RT + vincristine + isotretinoin
Andreiuolo F et al. (2019) [84]	N/A	2	15	2/0	2/0	N/A	At least 18	1: STR followed by RT + TMZ 1: GTR followed by RT + TMZ
Sasaki S et al. (2019) [60]	N/A	1	12	1/0	1/0	3	At least 12	1: STR x2. After progression, STR followed by TMZ + RT. After progression again, STR followed by RT + TMZ + BEV
Morris M et al. (2020) [63]	N/A	1	21	1/0	1/0	N/A	At least 10	1: Biopsy followed by RT + TMZ followed by lomustine + procarbazine + BEV
Rodriguez Gutierrez D et al. (2020) [124]	3–18 years	7	12.6	3/4	N/A	N/A	12	1: Biopsy followed by RT + TMZ 4: Surgery followed by RT + TMZ 1: Biopsy followed by RT + TMZ + BEV 1: Surgery followed by RT + TMZ + BEV
Onishi S et al. (2020) [73]	N/A	3	15	1/2	3/0	N/A	At least 18	1: GTR followed by chemotherapy (not specified) 1: GTR followed by RT + chemotherapy (not specified) 1: STR followed by RT + chemotherapy (not specified)
Crotty EE et al. (2020) [62]	<21 years	2	13.5	N/A	2/0	N/A	N/A	1: GTR followed by RT + TMZ followed by TMZ + irinotecan + BEV 1: STR followed by RT + TMZ followed by TMZ + irinotecan + BEV
Cheng Y et al. (2020) [61]	N/A	3	15	2/1	1/2	5	11	2: GTR followed by RT + TMZ 1: STR followed by RT + TMZ
Mohiuddin S et al. (2021) [114]	N/A	1	17	0/1	1/0	N/A	16	1: Biopsy followed by craniospinal RT + TMZ
Lim KY et al. (2021) [64]	N/A	4	28	1/3	3/1	N/A	24.5	1: Biopsy only 1: GTR followed by RT + TMZ 1: GTR followed by RT 1: GTR followed by vincristine + cyclophosphamide + prednisolone
Korshunov A et al. (2021) [72]	3–18 years	22	14	15/7	20/2	~12	~25	13: GTR followed by HIT-based protocol of RT (craniospinal RT + tumor bed boost) and cyclophosphamide + vincristine + methotrexate + carboplatin + etoposide (non-metastatic cases) or carboplatin + etoposide + intraventricular etoposide + cyclophosphamide + thiotepa + mesna 9: STR followed by HIT-based protocol as above
Picart T et al. (2021) [74]	>18 years	17	25	11/6	17/0	8.8	12.5	Combinations of treatments were not specified. 4: GTR 1: STR 12: Biopsy 13: RT + TMZ 1: RT only 1: RT + procarbazine + lomustine + vincristine 1: RT + TMZ
Wood MD et al. (2021) [117]	N/A	1	16	0/1	1/0	15	At least 15	1: GTR followed by RT + TMZ
Kalelioglu T et al. (2022) [121]	N/A	4	18	2/2	4/0	5	25	1: Biopsy followed by RT + clinical trial (not specified) 1: Surgery (not specified) followed by RT + TMZ 1: STR followed by RT + TMZ 1: GTR followed by RT + TMZ
Kitakami K et al. (2022) [125]	N/A	1	34	0/1	1/0	15	At least 20	1: Biopsy followed by RT + TMZ + BEV
Hu W et al. (2022) [51]	0–35 years	10	20.5	4/6	9/1	N/A	17.5	1: Biopsy followed by RT + TMZ 1: GTR followed by dianhydrodulcitol 4: GTR followed by RT + TMZ 3: STR followed by RT + chemotherapy (not specified) 1: STR followed by RT + TMZ + cisplatin
Kurokawa R et al. (2022) [120]	N/A	3	19	1/2	3/0	At least 10	At least 10	1: GTR followed by RT + TMZ + lomustine 1 GTR followed by RT + TMZ 1: GTR followed by RT + chemotherapy pending
Wang L et al. (2022) [66]	>18 years	30	24	11/19	26/4	10	14	2: STR followed by TMZ 3: Biopsy followed by RT + TMZ 13: STR followed by RT + TM 12: GTR followed by RT + TMZ
Yu N et al. (2023) [69]	N/A	1	19	0/1	1/0	N/A	N/A	1: Biopsy followed by RT + vincristine + cyclophosphamide + doxorubicin + mesna followed by ifosfamide + etoposide + mesna (initially diagnosed with sarcoma) followed by TMZ (after glioma diagnosis)
Lavrador JP et al. (2023) [75]	All	12	17.5	7/5	11/1	N/A	12	Combinations of treatments were not specified. 1: Biopsy 10: STR 1: GTR 1: Supratotal resection 9: RT + TMZ 1: RT only 1: TMZ only
Zhao Y et al. (2023) [116]	>18 years	1	27	0/1	1/0	13	16	1: STR followed by RT + TMZ
Cooley LD et al. (2023) [81]	0–17 years	2	12.5	1/1	2/0	N/A	16.5	2: STR followed by RT + chemotherapy (not specified)

Abbreviations: BEV, bevacizumab; EFS, event-free survival; GTR, gross total resection; HIT, HIT 2000 trial; OS, overall survival; PFS, progression-free survival; RT, radiation therapy; STR, subtotal resection; TMZ, temozolomide; ^a^ studies may have not had the data of interest for all patients. Numbers are based off of information that was available for patients; ^b^ some studies reported event-free survival, and some studies reported progression-free survival; ^c^ for studies that had patient-specific raw data but did not report event-free survival, progression-free survival, or overall survival, medians were calculated by reviewing raw data; ^d^ a tilde (~) denotes an approximation derived from visual inspection of Kaplan–Meier curves or of another figure; ^e^ “At least” indicates that a number of patients had yet to complete follow up, which would increase the median.

## Data Availability

No new data were created or analyzed in this study. Data sharing is not applicable to this article.

## Supplementary Materials

The following supporting information can be downloaded at: https://www.mdpi.com/article/10.3390/biomedicines11072002/s1, Table S1—Studies that created models to investigate the molecular pathways involved in tumorigenesis of H3-G34R/V mutant gliomas.
