# Supplementary material for: H3G34-Mutant Gliomas—A Review of Molecular Pathogenesis and Therapeutic Options
Source: Biomedicines. 2023 Jul 15;11(7):2002. doi: 10.3390/biomedicines11072002 (PMC10377039; doi:10.3390/biomedicines11072002)
Supplement: Supplementary file 1 [file biomedicines-11-02002-s001.zip › biomedicines-2485243-supplementary.pdf]

Table S1. Studies that created models to investigate the molecular pathways involved in tumorigenesis of H3-G34R/V mutant gliomas.

Study	Model(s)	Major Gene, Protein, Pathway, or Function Implicated	Brief Summary of Major Finding/Hypothesis
Bjerke L et al. (2013) [39]	(1) KNS42 (H3.3-G34V glioma patient-derived cells)<br>(2) Lentiviral transduction of H3.3-G34V into normal human astrocytes and transformation of human fetal glial cells	MYCN	H3.3-G34R/V may cause gliomagenesis through upregulation of MYCN.
Yadav RK et al. (2017) [36]	(1) S. pombe yeast with H3.3-G34R mutations	SETD2	The effects of the H3.3-G34R mutation involve more than simple disruption of SETD2 binding. G34R mutant cells exhibited genomic instability, sensitivity to DNA damage/replication stress, and defective homologous recombination-directed repair.
Zhang Y et al. (2017) [27]	(1) *E. coli* (for expression of human SETD2)<br>(2) Mutant fission yeast strains generated by the PCR-based module method (for G34R/V)	SETD2	H3.3 binds within a narrow substrate channel of SETD2, and mutations result in steric clashing and decreased binding. H3.3-G34R/V mutations thus result in decreased H3K36me3 in cis.
Fang J et al. (2018) [28]	(1) HEK293 engineered to express H3.3-G34R/V mutant histones (transfection or mutagenesis technique not specified)<br>(2) KNS42 (H3.3-G34V glioma patient-derived cells)<br>(3) SF188 engineered to express ectopic H3.3-G34R/V mutant histones (transfection or mutagenesis technique not specified)	SETD2<br>NSD1 and NSD2<br>MutSα	The H3.3-G34R/V mutant histones result in decreased activity of SETD2, NSD1, and NSD2, inhibiting methylation of H3.3K36.<br><br>MutSα, a DNA mismatch repair enzyme, recognizes H3K36me3, and since H3.3-G34R/V inhibits trimethylation of H3K36, MutSα is unable to bind to chromatin harboring the mutant histone.<br><br>H3.3-G34V glioma cells exhibited a high mutation rate that was unrelated to homologous recombination-directed DNA repair.
Lim J et al. (2018) [38]	(1) Zinc finger targeting methodology to create H3.3-G34R and subsequent transfection of HEK293	HIST1H2AC<br>HIST3H2A	The protein-protein interaction score of HIST1H2AC and HIST3H2A with H3.3-G34R in HEK293 cells is high and unique to the G34R mutation.
Shastrula PK et al. (2018) [37]	(1) KNS42 (H3.3-G34V glioma patient-derived cells) subjected to Lentiviral transfection with Rpp29 shRNA<br>(2) Addition of an shRNA-resistant Rpp29 to (1) generated by QuikChange Lightning site-directed mutagenesis	Rpp29	H3.3-G34R binds to Rpp29 with significantly lower affinity than H3.3-wt.<br><br>Knocking out Rpp29 in resulted in increased H3.3 at the promoters and gene bodies of DLX-5/6 and MYCN, genes associated with G34 mutant glioma, suggesting Rpp29 may repress H3.3 incorporation in the chromatin of transcriptionally active genes. The mRNA levels of these genes approximately doubled with Rpp29-ko. Rpp29-ko does not completely impair translation.<br><br>Rpp29-ko resulted in increases of both H3K36me3 and H3K4me3.<br><br>Generation of shRNA-resistant Rpp29 demonstrated that Rpp29 is directly responsible for the change in H3.3 incorporation. In addition, Rpp29-ko impacted histone post-translational modifications and increased the number of unmodified histones from multiple different histone families, including H3 (and its variant H3.3). Rpp29-ko decreased H3K9me3, H3.1K27me3, and H3.2K27me3. It also resulted in other modifications consistent with increasing transcription. Given its effects on H3K27, it may interact with or regulate PRC2. Regarding H3.3, Rpp29-ko resulted in decreased H3.3K27me3 and increased H3.3K36me1.
Voon HPJ et al. (2018) [33]	(1) Cre-recombinase to create mouse embryonic cells with the H3f3a-G34R mutation at its original locus	KDM4 histone lysine demethylases (A/B/C)	H3.3-G34R binds and inhibits KDM4 family histone demethylases, which contributes to increased H3K9me3 and H3K36me3. This may be how G34R exerts its effects in a dominant-negative manner.

Lowe BR et al. (2019) [20]	(1) Fission yeast	H3.3-G34R	The substituted lysine in H3.3-G34R is not methylated in vitro.
Chen CCL et al. (2020) [57]	(1) CRISPR/Cas9 vectors (for TP53 mutations), and the PiggyBac transposon system (with ATRX shRNA) were utilized to transduce mouse NPCs; QuikChange Lightning site-directed mutagenesis for H3.3-G34R and PDGFRA-D842V mutants<br>(2) CRISPR/Cas9 to remove G34R and knock-in H3.3-wt in HSJD-GBM002 (H3.3-G3R glioma patient-derived cells)<br>(3) CRISPR/Cas9 to remove G34V in KNS42 (H3.3-G34V glioma patient-derived cells)	PDGFRA and MAPK/ERK<br>GSX2 and DLX1/2	PDGFRA was mutated, with increased expression, in 44% of newly diagnosed G34 mutant gliomas and in 81% of recurrent cases. PDGFRA amplification was an uncommonly observed mutation (13% of PDGFRA mutations). Post-translational modification of cis regulatory elements as well as changes in chromatin conformation can increase PDGFRA expression in G34 mutant gliomas. PDGFRA activates MAPK/ERK signaling and results in clonogenicity and astrocytic expansion at the expense of neurons. H3.3-G34R/V may become dispensable following acquisition of PDGFRA overexpression.<br><br>H3.3-G34R/V led to increased H3K27me3 at many promoters, including those of mature forebrain transcription factors. GSX2 and DLX1/2, which are key transcription factors of interneuron specification and inhibition of oligodendrocyte specification, are preserved in G34 mutant gliomas. These genes have higher levels of H3K27ac and lack H3K27me3. GSX2 also forms a chromatin loop that is in close proximity to PDGFRA (both on chromosome 5 in mice) that may allow for overexpression of PDGFRA. G34 mutant glioma cells may arise from prenatal interneuron progenitors.
Huang TY et al. (2020) [30]	(1) Lentiviral transfection of KNS42 (H3.3-G34V glioma patient-derived cells) with inducible anti-H3F3A siRNA<br>(2) Lentiviral transfection of normal human astrocytes with H3.3-G34V mutation	SOX2<br>Cellular neuron projection morphogenesis<br>Neuronal differentiation	The H3.3-G34V mutation decreases H3K36me3 in cis. H3.3-G34V results in co-enrichment of H3.3-wt, which may be expressed by H3F3B. The G34V mutation additionally results in increased H3K27me3. Co-enrichment by H3.3-wt occurred at SOX2 in cells harboring the H3.3-G34R/V mutation. Genes differentially upregulated serve functions related to cellular neuron projection morphogenesis and neuronal differentiation.
Jain SU et al. (2020) [26]	(1) Lentiviral transfection of HEK293T with H3.3-G34R/V	SETD2<br>PRC2	H3.3-G34R/V mutations decrease levels of H3K36me3 and increase levels of H3K27me3 in cis. The mutation results in the former by blocking SETD2 binding and activity. However, the G34 mutations still allow PRC2 binding, and PRC2 would normally be blocked by H3K36me3. Thus, G34R/V mutations indirectly result in increased PRC2 activity (and H3K27me3) by decreasing H3K36me3.
Jiao F et al. (2020) [100]	(1) E. coli (RACK7 domains and H3.3 histones including H3.3-G34R/V mutants) and Baculovirus transfection of Sf9 for full-length RACK7<br>(2) SJ-HGGx6c and SJ-HGGx42c cell lines (H3.3-G34R glioma patient-derived)<br>(3) CRISPR/Cas9 knock-in of cell lines in (2) with H3.3-wt	RACK7<br>MHC II genes, their chaperones, CD74, and CIITA	RACK7 binds to H3.3-G34R, downregulating expression of junction proteins, vesicle-related genes, and CIITA. CIITA is known to upregulate expression of MHC II genes, their chaperones, and CD74, and thus its downregulation also leads to downregulation of MHC II, which plays a role in adaptive immunity and possibly antitumor immunity. RACK7 knock-out or H3.3-wt knock-in resulted in decreased glioma cell migration and invasion.
Bressan RB et al. (2021) [42]	(1) The PiggyBac transposon system was utilized for H3.3-G34R and PDGFRA amplification mutations; CRISPR/Cas9 for TP53; transfected human forebrain vs hindbrain fetal NSCs<br>(2) CRISPR/Cas9 to delete the G34R mutation in pGBM002 (H3.3-G34R glioma patient-derived cells)<br>(3) CRISPR/Cas9 knock out FOXG1 in pGBM002 (H3.3-G34R glioma patient-derived cells), aGBM7, and aGBM313 (the latter two are H3.3-wt glioma patient-derived cells)	ZMYND11<br>FOXG1 and CDKN1A/p21	The H3.3-G34R mutation, when combined with PDGFRA amplification and TP53-ko, increased clonogenicity and proliferation of forebrain NSCs. These mutations rendered hindbrain NSCs cytostatic.<br><br>CRISPR/Cas9 deletion of the H3.3-G34R allele in pGBM002 resulted in cells that resembled the triple mutant forebrain NSCs in their transcriptional expression of SOX2 and NESTIN. Knockout of H3.3-G34R mutations reduced proliferation and clonogenicity. Engineered expression of H3.3-G34R reversed this. It was noted that the G34R-ko cells only had marginal reduction of MYCN.<br><br>Key upregulated genes in the triple mutant forebrain NSCs included neural progenitor markers OLIG2 and SOX3 and forebrain-specific transcription-factors DMRTA2, EMX2, NR2F1, and HIVEP2. Single mutant H3.3-G34R forebrain NSCs did not exhibit widespread transcriptomic changes, suggesting additional mutations are needed for gliomagenesis.

			H3.3-G34R was enriched at active promoters and gene bodies, which both correlated with higher levels of H3K4me3 and H3K36me3. H3.3-G34R incorporation was increased at genes with high transcription, and the genes differentially downregulated in G34R-ko cells were genes with increased H3.3-G34R enrichment (including forebrain-related genes).<br><br>H3.3-G34R has decreased affinity for ZMYND11, a transcription repressor that reads H3K36me3 and alters elongation and splicing of genes.<br><br>Deletion of FOXG1, a transcription factor critical to forebrain specification, by CRISPR/Cas9 reduced H3.3-G34R mutant glioma cell proliferation and eliminated tumorigenesis. This deletion also resulted in upregulation of p21, which is a known contributor to senescence. Of note, the cytostasis of G34R mutant hindbrain NSCs is associated with activation of p21 as well. Thus, FOXG1 may be important in H3.3-G34R mutant gliomagenesis by downregulating p21-mediated senescence and allowing transcription of forebrain-related genes, thus acting in a regional-dependent manner.
Funato K et al. (2021) [44]	(1) Human ESCs were differentiated to interneuron ventral forebrain NPCs and hindbrain NPCs, and QuikChange Site-Directed Mutagenesis was utilized to introduce H3.3-G34R mutations while CRISPR/Cas9 was utilized to create TP53-ko and ATRX-ko mutations in both cell lines.<br>(2) Addition of N-Myc to (1) (method unclear) and then transplanted into immunodeficient mice<br>(3) Lentiviral transfection of model (1) with the H3.3-K36R mutation<br>(4) SJHGGx6 and HSJD002 cell lines (G34R mutants)<br>(5) Lentiviral transfection of model (1) with NOTCH2NLB for overexpression studies<br>(6) Transfection of HSJD002 with NOTCH2NL shRNA to create NOTCH2NL-ko	ASCL1<br>ZMYND11<br>NOTCH2NL	The authors optimized an NPC differentiation protocol and developed an H3.3-G34R mutant glioma model based upon human ESCs.<br><br>Forebrain NPCs with ATRX-ko, TP53-ko, and G34R mutations formed an increased number of rosette structures (which are normally only transiently formed during differentiation) by day 46 of differentiation and more frequently expressed PLZF, a primitive neuroepithelial marker. Hindbrain NPCs did not behave similarly.<br><br>N-Myc introduction to ventral forebrain NPC triple mutants did allow for tumor formation in vivo in xenograft models. Hindbrain NPCs did not form tumors. The tumors created in this model demonstrated low CpG methylation levels and alternative lengthening of telomeres, similar to patient-derived H3.3-G34R mutant glioma cells.<br><br>Triple mutant forebrain NPCs shared similar RNA sequencing patterns with patient-derived H3.3-G34R mutant cells from the SJHGGx6 and HSJD002 lines. The forebrain NPCs were found to have upregulation of genes associated with stem cell population maintenance and downregulation of ASCL1, a transcription factor essential for neuronal differentiation.<br><br>Triple mutant forebrain NPCs demonstrated an increase in SOX2 and a decrease in Tuj1, suggesting a propensity toward stem cell maintenance instead of neuronal differentiation. These cells also demonstrated increased HES1 and HEYL, suggesting the involvement of the Notch pathway. Analysis of alternative splicing demonstrated that the triple mutant forebrain NPCs exhibited suppressed intron retention of several Notch pathway genes, including NOTCH2NL (which is increased, along with HES1, in H3.3-G34R mutant patient tumors). The decrease in intron retention was mapped to the last intron of NOTCH2NL and resulted in increased functionally spliced NOTCH2NL mRNA transcripts. Analysis of this site also demonstrated decreased H3K36me3 and its reader ZMYND11. Overexpression of NOTCH2NL increased forebrain NPC proliferation and decreased hindbrain NPC proliferation. Knockdown of NOTCH2NL with shRNA in H3.3-G34R mutant lines decreased cell proliferation and decreased the tumor burden of the triple mutants.<br><br>The NOTCH2NL locus was amplified in 44% of H3.3-G34R

			mutant gliomas, suggesting upregulation of NOTCH2NL could be controlled by two separate mechanisms.
Lemon LD et al. (2022) [31]	(1) Saccharomyces cerevisiae yeast with various histone mutations (including H3.3-G34R/V)	H3.3-G34R/V	Although H3.3-G34R/V mutant yeast had an increased double time, they achieved greater biomass than control strains.<br><br>H3.3-G34R/V mutations decreased H3K36me3 and altered sensitivity to disruption of different cellular pathways. H3.3-G34R/V mutants were sensitive to formamide (inhibits RNA metabolism) and hydroxyurea (impairs DNA synthesis).
Lowe BR et al. (2021) [45]	(1) Fission yeast with H3.3-G34R/V mutations	Gcn5<br>Homologous recombination-directed DNA repair	H3K36me3 is decreased in H3.3-G34R/V mutants. H3K36ac is decreased in cis in H3.3-G34R mutants but not G34V. Gcn5-catalyzed acetylation of H3K36 is dramatically reduced on histones with G34R and simply slowed on histones with G34V.<br><br>H3.3-G34R mutant cells are sensitive to replicative stress, and G34V cells are sensitive to ionizing radiation.<br><br>The H3.3-G34R mutation inhibits homologous recombination-directed DNA repair and results in a mutator phenotype, while the H3.3-G34V mutation did not inhibit homologous recombination-directed repair or result in the same degree of genomic instability.<br><br>H3.3-G34R mutant cells accumulate Sgo2 at subtelomeric regions, which form knobs and decrease transcription and thus expression of genes in these regions. This may be related to decreased levels of H3K36ac.
Sweha SR et al. (2021) [48]	(1) H3.3-G34R mutant immortal mouse NSCs<br>(2) KNS42 (H3.3-G34V glioma patient-derived cells)<br>(3) CHOP-GBM001 and HSJD-GBM002 (H3.3-G34R glioma patient-derived cells)<br>(4) The PiggyBac transposon system was utilized in injection of the TP53, PDGFRA, and H3.3-G34R mutations into the lateral ventricle of E13.5 CD-1 mice<br>(5) SJHGGx6-c and SJHGGx42-c (H3.3-G34R glioma patient-derived cells)<br>(6) OPGB-GBM-001 (H3.3-G34R glioma patient-derived cells)<br>(7) KNS42 cells injected into the flank of athymic nude mice<br>(8) KNS42 cells injected into the cortex of NSG mice<br>(9) Cells from (4) and SJHGGx42-c implanted into NSG mice	LIF, JAK, and STAT3	H3.3-G34R mutant mouse NSCs exhibited upregulation of genes involved in chemokine and cytokine signaling as well as the JAK/STAT pathway. The LIF gene was highly upregulated. Levels of H3.3-G34R/V correlated with the degree of H3K36me3 enrichment at the LIF locus. The chromatin at the LIF locus appeared to be in an activated transcription state. The LIF promoter was additionally hypomethylated. The H3.3-G34R mutation results in increased H3K36me3 at the gene body of LIF, increased H3K27ac and H3K4me3 at its promoter, and decreased H3K27me3.<br><br>LIF mRNA and the downstream product of LIF's catalyzed reaction, phosphorylated Stat3 is increased in G34 mutant glioma cells.<br><br>Downregulation of LIF resulted in decreased pStat3 and total Stat3; it also resulted in cell death. CRISPR/Cas9-mediated knock out of STAT3 resulted in changes in genes involved in cell differentiation and motility, apoptosis and cell death, and neuronal differentiation. STAT3-ko KNS42 cells had increased survival and lower tumor burden relative to STAT3-wt cells.<br><br>Stattic and WP1066, small-molecule inhibitors of STAT3, demonstrated growth inhibition and toxicity of G34 mutant glioma cells as well as decreased tumor volumes. WP1066 increased survival of mice with H3.3-G34R/V mutant tumors and, in combination with radiation therapy, may be more effective than radiation therapy alone.
Udugama M et al. (2021) [55]	(1) CRISPR/Cas9 to knock out ATRX, TP53, TERT, and KDM4B in mouse embryonic cell lines; Cre-recombinase to introduce the H3.3-G34R mutation	KDM4B<br>ATRX<br>Alternative Lengthening of Telomeres	The H3.3-G34R mutation binds KDM4B, the most prevalent telomeric demethylase, and regulates chromatin accessibility at the telomeres. Loss of both ATRX and KDM4B (the former via mutation and the latter via H3.3-G34R binding and inhibition) allows for activation of alternative lengthening of telomeres.
Haase S et al. (2022) [32]	(1) Sleeping Beauty transposon system to create mouse neural precursor cells with H3.3-G34R and constitutively active NRAS	DNA damage repair<br>cGAS/STING	H3.3-G34R mutant glioma cells exhibited decreased transcription of genes associated with DNA repair, the cell cycle, and chromatin structure. They also exhibited decreased MGMT expression.<br><br>H3.3-G34R mutant glioma cells demonstrated decreased

	mutations as well as shRNA against P53 and ATRX<br>(2) Transfection of SJ-GBM2 (human patient derived glioblastoma cells) to express H3.3-G34R<br>(3) OPBG-GBM001 and HSJD-GBM002 (human patient derived H3.3-G34R mutant glioma cells)<br>(4) Sleeping Beauty transposon to create mouse neural precursor cells with H3.3-G34R and PDGFRA mutations in addition to Cdkn2a deletion and both ATRX and P53 downregulation.		capabilities to carry out both homologous recombination repair and nonhomologous end-joining. This is due to decreased levels of DNA repair and cell cycle proteins as well as their active forms (which require posttranslational modifications). H3.3-G34R mutant glioma cells were more sensitive to ionizing radiation.<br><br>Pamiparib, a PARPi that inhibits DNA damage repair, combined with ionizing radiation were more effective in H3.3-G34R mutants than in H3.3-wt counterparts. H3.3-G34R mutants were also more sensitive to AZD7762, a cell cycle checkpoint kinase 1/2 inhibitor. Sensitivity to temozolomide was likewise greater in the G34R mutants.<br><br>H3.3-G34R mutants demonstrated greater genomic instability and had an increased number of genomic rearrangements.<br><br>Mice that survived H3.3-G34R mutant gliomas after undergoing radiation therapy demonstrated decreased formation of tumors upon subsequent inoculation; 20% formed tumors, suggesting there is adaptive immune memory. H3.3-G34R mutant gliomas treated with ionizing radiation release interferon-beta in a cGAS/STING pathway dependent manner. Additionally, the cGAS/STING pathway may be involved with release of damage-associated molecular patterns into the tumor microenvironment, facilitating immunologic cell death and contributing to antitumor memory.<br><br>Pamiparib and radiation therapy resulted in long-term survival in 60% of mice implanted with (1), whereas AZD7762 and radiation therapy resulted in long-term survival in 40%. While pamiparib was effective without radiation therapy, AZD7762 was not. CD8-ko mice did not survive long-term even with treatment, suggesting that the adaptive immune system plays a key role in the efficacy of ionizing radiation and pamiparib. The STING agonist diABZI improved long-term survival in the radiation only group (and did not affect survival in the other groups). The STING antagonist H151 eliminated the efficacy of pamiparib and radiation therapy combination treatment, reducing survival to even less than that of the non-treated group.<br><br>Veliparib, a PARPi, with high blood-brain barrier permeability (previously evaluated in ACNS1721) was less potent than pamiparib and did not increase the efficacy of radiation therapy.
Siddaway R et al. (2022) [34]	(1) Lentiviral transfection of HEK293 cells with the H3.3-G34R mutation<br>(2) 7316-158 cells (H3.3-G34R glioma patient derived cells)	Histone modifying proteins<br>DNA damage repair<br>Mitochondrial metabolism<br>SPEN and NOTCH<br>Cryptic transcription	Compared to H3.3-wt, H3.3-G34R generally lost interactions with proteins, and comparisons mentioned therein are compared to H3.3-wt. The G34R mutation resulted in decreased interaction with NSD1-3 (which dimethylate H3K36) and PRC2. PRC2 may bind to H3.3-G34R for a shorter period of time.<br><br>H3.3-G34R lost interactions with a large number of proteins that catalyze post-translational modifications of histones, including methylases, demethylases, acetylases, and deacetylases, suggesting that there may be even more epigenetic effects of this mutation that have yet to be identified. There were also decreased interactions with DNA damage repair proteins.<br><br>The pathways significantly enriched in this mutation are involved in metabolism. Some of the proteins enriched in H3.3-G34R localized to mitochondria. Staining of mitochondria revealed colocalization of a minority of H3.3-G34R (the majority of which was in the nucleus) with mitochondria.<br><br>H3.3-G34R had decreased associations with DNMT1 and DNMT3A, which are DNA methyltransferases. Compared to H3.3-wt tumors, H3.3-G34R tumors had significant hypomethylation; of the >10,000 differentially methylated regions

			between the two, 88% were relatively hypomethylated in G34R mutants. The genes affected are involved in metabolism and differentiation.<br><br>H3.3-G34R had decreased interactions with SPEN, a repressor of NOTCH, and H3.3-G34R was associated with increased cryptic transcription.<br><br>H3.3-G34R was associated with decreased H3K36me2/3 in cis, but H3K27me3 and H3K9me3 were increased on both H3.3-G34R and its H3 partner. SUV39H2 and EHMT2 activity is increased on H3.3-G34R relative to H3.3-K27M in catalyzing trimethylation of H3K9. shRNA targeting SUV39H1, SUV39H2, EHMT1, and EHMT2 decreased cell viability of (2), suggesting H3K9 methylation plays a key role in G34R mutant glioma cell viability.<br><br>Similarly, OTS186935, an inhibitor of SUV39H2, and chaetocin, an inhibitor of SUV39H1/2 and EHMT1/2, had a lower IC50 in oncohistone cell lines compared to H3.3-wt cell lines. This again suggests that H3K9 may be a potential therapeutic target.
Abdallah AS et al. (2023) [56]	(1) RCAS/tv-a utilized to overexpress Cre, PDGF-A, and the H3.3-G34R mutation in the frontal cortex (via injection of DF1 cells with RCAS virus between postnatal days 3-5) of mice with floxed P53 and either floxed ATRX or wt ATRX	H3.3-G34R<br>ATRX	Note: Olig2 was highly expressed in most of the samples.<br><br>H3.3-G34R mutation status did not affect tumor incidence or latency in. However, ATRX loss increased tumor latency in H3.3-wt (not H3.3-G34R mutant) mice.<br><br>ATRX-ko mice had lower rates of ependymal cell differentiation.<br><br>ATRX-ko had a greater effect on transcriptomics than the H3.3-G34R mutation. ATRX-ko H3.3-G34R mutant mice had upregulation of HOXA2, HOXA3, HOXA5, and HOXA7. Additionally, there was upregulation of cell proliferation and metabolism genes. ATRX-ko and H3.3-G34R resulted in upregulation of neuronal markers.<br><br>COL12A and NEFL were upregulated in ATRX-ko as well as in a dataset of human-derived H3.3-G34R mutant glioma cells. Of the differentially expressed genes between ATRX-ko vs ATRX-wt, five (COL5A1, COL6A2, KHDC8A, PDGFD, and PGM5) had concordant overexpression in G34R mutant (vs H3.3-wt), suggesting ATRX-ko may play a key role in the altered transcription of H3.3-G34R mutant gliomas.
Khazaei S et al. (2023) [43]	(1) CRISRP/Cas9 utilized to introduce H3.3-G34R/V/W mutations into mice 2-cell or 4-cell stage embryos which were bred to adulthood and backcrossed with H3.3-wt mice (C57BL/6J and B6C3F1/Crl strains) to create H3.3-G34R/V heterozygous mice<br>(2) CRISP/Cas9 utilized to introduce an inducible (lox) H3.3-G34R mutation into mice which were then crossed with mice expressing Emx1-Cre (excitatory NPCs and astrocytes in the dorsal neuroepithelium) and Foxg1-Cre (excitatory NPCs in the dorsal forebrain and inhibitory NPCs in the ventral forebrain) in order to induce the H3.3-G34R mutation only in neurons	ASHL1 and DNMT3A<br>Complement	The H3.3-G34R mutation resulted in severe neurologic phenotypes while the G34W mutation resulted in mesenchymal-related defects. G34V mutant mice were in the middle of the spectrum between G34R and G34W.<br><br>H3.3-G34R mice developed ataxia and neuromuscular defects after 3 months. They demonstrated progressive loss of cerebellar foliation with age, which was seen to a lesser degree in G34V mice. In G34R mice, this was accompanied by a decrease in number of Purkinje cells at age 1 year. Additionally, H3.3-G34R mice exhibited higher levels of microglia and decreased levels of L5/L6 glutaminergic neurons.<br><br>While there was transcriptional dysregulation evident in the brain of a 7-day-old mouse of model (1), dysregulation was present to a greater degree in the brain of a 10-week-old mouse.<br><br>G34R mutant mice accumulated microglia (demonstrated to be disease-associated) and reactive astrocytes in the deeper cerebral cortical layers.<br><br>G34R mutant mice demonstrated the greatest decrease in levels of K36me2/3 in cis. BioID demonstrated that H3.3-G34R had decreased interactions with ASHL1 (H3K36 dimethyltransferase),

			ZMYND11, DNMT3A, and DNMT1.<br><br>The decreased binding of DNMT3A, which recognizes H3K36me2, resulted in DNA hypomethylation (CH more so than CG) in H3.3-G34R mutants, preferentially at intergenic regions. Some CpG islands were noted to be hypermethylated. As DNA methylation is antagonistic to (and does not co-occur with) H3K27me3, the change in DNMT3A distribution is thus thought to result in altered regulation of genes that are normally under the control of PRC2/H3K27me3.<br><br>Genes upregulated due to hypomethylation in G34R mutants include genes involved in complement (C1QA/B/C, C3, and C4A/B) and cytokine signaling (CCL3/4). Some neuronal genes (NPTX2 and NEFM) were found to be methylated and downregulated. The former results in inappropriate continued complement signaling, which is thought to normally serve a key role in recruiting microglia and astrocytes to neurons during early development.<br><br>Development of model (2) demonstrated that neuronal expression of H3.3-G34R resulted in complement activation, inflammation with upregulation of microglia and reactive astrocytes, and loss of deeper cortical neurons.
McNicholas M et al. (2023) [58]	(1) CRISPR/Cas9 was utilized to introduce TP53-ko, and the PiggyBac transposon system was utilized to introduce the H3.3-G34R mutation and ATRX shRNA as well as either PDGFRA-wt, PDGFRA-D842V, or PDGFRA-C235Y into the dorsal pallium (cortex) or ventral pallium (ganglionic eminence) of mice at age embryonic day 12.5 via in utero electroporation<br>(2) Gliomasphere cell lines generated from (1)	PDGFRA	Note: GPAP, GPAD, and GPAC refer to mice with the H3.3-G34R mutation, TP53 mutation, shATRX, and either PDGFRA-wt, PDGFRA-D842V, or PDGFRA-C235Y respectively. GPAP was intended to represent H3.3-G34R mutant gliomas with PDGFRA amplification.<br><br>GPAP, GPAD, and GPAC all induced tumor formation (although GPAD had 100% penetrance and the others had ~55% penetrance). Without PDGFRA amplification or mutations, tumorigenesis did not occur. Introduction of PDGFRA mutant vectors with H3.3-wt into the ganglionic eminences resulted in a large degree of mortality, with only 34-38% surviving to weaning. This lethality was abrogated by co-delivery of PDGFRA mutations with the H3.3-G34R mutant.<br><br>GPAC-derived (2) cells were not sensitive to the PDGFRA inhibitor avapritinib but were very sensitive to the FGFR inhibitor infigratinib.<br><br>GPAD tumors had the highest mitotic index of the three. High levels of H3K27me3 were noted in all three.<br><br>Grafting of (2) into the striatum of C57BL/6J mice successfully produced tumors. Of note, attempting to introduce the mutations of (1) into ganglionic eminence NSCs in vitro did not produce an engraftable tumor cell line, suggesting that introduction of these mutations into an immunocompetent environment in vivo is necessary to create an engraftable tumor cell line.

Abbreviations: DNA, deoxyribonucleic acid; ESC, embryonic stem cell; ko, knock-out; L5, layer 5; L6, layer 6; mRNA, messenger RNA; NPC, neural progenitor cell; NSC, neural stem cell; PARPi, poly ADP ribose polymerase inhibitor; PCR, polymerase chain reaction; RCAS, Replication-Competent Avian sarcoma leukosis virus long-terminal repeat with Splice acceptor; RNA, ribonucleic acid; shRNA, short hairpin RNA; siRNA, silencing RNA; wt, wild-type.
